# A simple approach for rapid and cost-effective quantification of extracellular vesicles using a fluorescence polarization technique

**DOI:** 10.1186/s13036-019-0160-9

**Published:** 2019-04-16

**Authors:** Kalishwaralal Kalimuthu, Woo Young Kwon, Ki Soo Park

**Affiliations:** Department of Biological Engineering, College of Engineering, Konkuk University, Seoul, 05029 Republic of Korea

**Keywords:** Biosensor, Extracellular vesicles, Fluorescence polarization, Quantification

## Abstract

Extracellular vesicles (EVs) are membrane-bound phospholipid vesicles actively secreted by all cells. As they carry specific markers expressed by their parental cells, EVs are utilized to identify specific cells via liquid biopsy. To facilitate EV-based clinical diagnosis, a fast and reliable method to count EVs is critical. We developed a method for rapid and cost-effective quantification of EVs which relies on the fluorescence polarization (FP) detection of lipophilic fluorescein probe, 5-dodecanoylamino fluorescein (C12-FAM). The alkyl tail of C12-FAM is specifically incorporated into the EVs, producing high FP values due to a slow diffusional motion. We quantified EVs derived from two cell lines, HT29 and TCMK1 using the new strategy, with good sensitivity that was at par with the commercial method. The new method involves minimal complexity and hands-on time. In addition, FP signaling is inherently ratiometric and is robust against environmental noise.

**Electronic supplementary material:**

The online version of this article (10.1186/s13036-019-0160-9) contains supplementary material, which is available to authorized users.

## Introduction

An emerging new approach to disease diagnosis and treatment monitoring is to exploit circulating biomarkers that can be repeatedly and conveniently obtained with minimal complications [1, 2]. This technique, called “liquid biopsy” has emerged as the next-generation, diagnostic and monitoring tool for diseases such as cancers, with the benefit of being less-invasive compared to traditional biopsy [2, 3] In particular, extracellular vesicles (EVs) (30–200 nm in diameter) that are secreted by their parental cells and circulating in the biological fluids are of special interest as they carry genomic and proteomic signatures of their parental cells [4]. A growing number of studies have demonstrated that EVs function as reliable surrogates of their original cells for non-invasive diagnosis of cancers [5–7].

Till now, many researchers have devised different strategies for the streamlined analysis of EV biomarkers such as proteins and nucleic acids [8]. For example, Jiang et al. proposed a colorimetric strategy for the detection of EV surface proteins, which utilizes gold nanoparticles complexed with a panel of aptamers [9]. In the presence of specific EVs, aptamers that have the affinity to EV protein markers are released from gold nanoparticles to generate specific colorimetric patterns. In another study, Shao et al. developed a microfluidic platform, termed immuno-magnetic exosome RNA (iMER) which consists of three functional modules: EV isolation, RNA extraction, and real-time PCR to analyze mRNA targets inside EVs related to drug treatment efficacy [10].

Despite significant advances in the analysis of EV biomarkers, technical challenges still remain in the quantification of EVs, which is key for the downstream analysis of EV biomarkers. In addition, it is reported that the level of EVs may themselves be used for early diagnosis of cancer or cancer relapses, which has been evidenced by the fact that EVs are secreted from cancer cells at an increased rate, compared to normal cells [11–13]. To date, direct particle counting systems, including nanoparticle tracking analysis (NTA), flow cytometry, and tunable resistive pulse sensing have been utilized for quantifying EVs [14]. However, the requirement of sophisticated technical skills and special and bulky instruments that are rarely available in most laboratories greatly limits their wide-spread and practical applications [15–18]. As a promising alternative, System Biosciences commercializes a kit named “EXOCET exosome quantification kit”, which relies on the acetylcholinesterase (AChE) enriched within EVs and confirms that EV counts measured by AChE assay are in accordance with that calculated by NTA [19–21]. In addition, it has been reported that this kit works well in different types of EVs derived from cancer cells, stem cells and even serum [20, 22]. Although the commercial kit shortens the total assay time and has a good assay performance, it still entails the tedious steps including EV lysis, centrifugation, and enzyme reaction to generate the colorimetric signals. More importantly, it is quite expensive due to the proprietary rights (~$6 for a single assay). Therefore, there is a high demand for simple and cost-effective methods to reliably count EVs.

In this study, we devised a general approach to quantify EVs that do not require the expensive reagents and washing steps. Our system relies on the fluorescence polarization (FP) detection of lipophilic fluorescein probe, 5-dodecanoylamino fluorescein (C12-FAM), which has been used for the determination of critical micelle concentration value of surfactants [23]. As EVs like cells are surrounded by a phospholipid bilayer membrane, we expect that C12-FAM composed of an aliphatic, alkyl tail and the fluorophore would be inserted onto the EVs. As a result, the effective molecular volume of C12-FAM in the presence of EVs would become significantly increased, compared to that of C12-FAM only, leading to the high FP values due to the slow rotational speed. Using this method, we successfully quantified EVs derived from cancer and normal cells and compared the values with the ones derived by the commercial method. In addition, we confirmed that cancer cells secrete EVs in an increased rate, compared to normal cells. Our system is quite beneficial for the practical applications because all reactions take place in a single tube without any washing steps, which achieves a “mix-and-read” assay and it is robust against environmental noise as FP signaling is inherently ratiometric.

## Results and discussion

### FP-based quantification of EVs

**Scheme 1:**
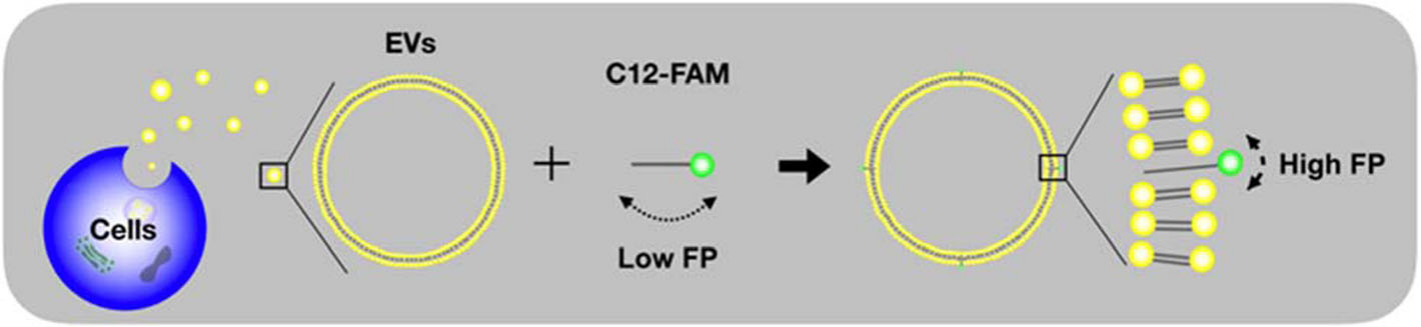
Illustration (not drawn to scale) of FP-based EV quantification method

The conceptual design of the EV quantification method is illustrated in Scheme 1, which utilizes C12-FAM as the key detection component. Following the isolation of EVs secreted from parental cells, they are incubated with C12-FAM that contains two regions: (i) the fluorophore that generates FP values, and (ii) the lipophilic tail composed of alkyl groups that anchor onto EV membranes (See Materials and Methods for details). The lipophilic tail of C12-FAM is inserted into the phospholipid bilayer of EVs and thus it assumes high FP (FP), compared to the one in the absence of EVs. The entire process can be performed in a single tube without separation or washing steps.

### Characterization of EVs

**Fig. 1:**
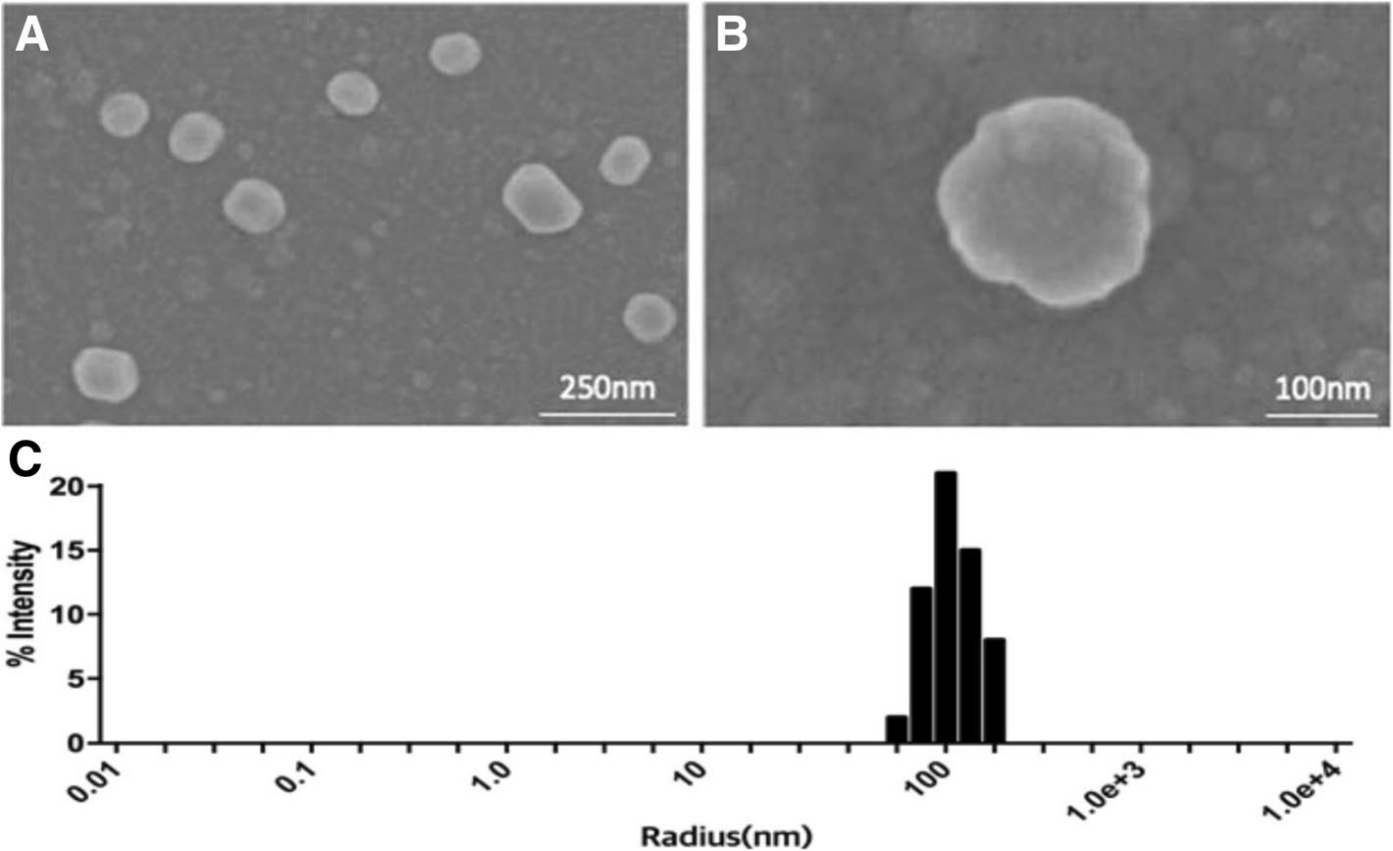
Characterization of EVs derived from HT-29. **a** and **b** SEM image of EVs. **c** Size distribution of EVs

As a proof-of-concept, we applied the developed system to count EVs secreted from cancer cells, HT-29. First, we characterized the isolated EVs by scanning electron microscopy (SEM) and dynamic light scattering (DLS) analyses. Even though the ultracentrifugation is widely utilized for the isolation of EVs and does not involve the chemical precipitants, it requires a bulky instrument and entails the problems of long preparation time and low purity [24]. Thus, in this experiment, we chose the chemical precipitation method (EXO-Quick-TC, Systems Biosciences), which is simple and commercially available. As shown in Fig. 1, the EVs isolated from HT-29 exhibited a round morphology with uniform, size distribution (ca. 200 nm), which is consistent with that reported in the literature [24, 25].

### Quantification of EVs

**Fig. 2:**
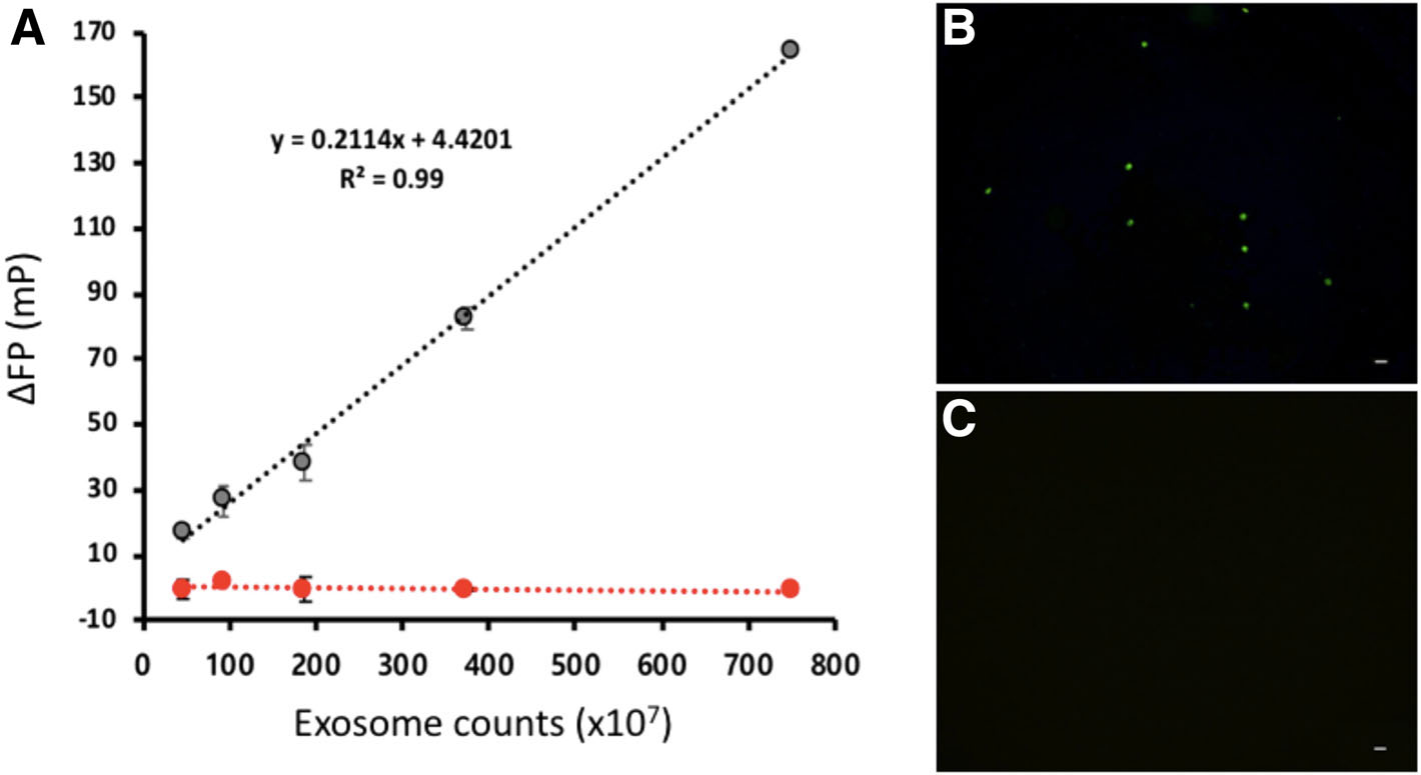
Quantification of HT-29 EVs. **a** The calibration curve of standard EVs (Gray: C12-FAM and Red: FAM). **b** and **c** The fluorescence microscopy images (scale bar = 10 μm) obtained from HT-29 EVs with C12-FAM (**b**) and FAM (**c**). EVs are in clusters as well as single vesicles and the clusters that are effectively stained with C12-FAM are visualized under the fluorescence microscopy [30]

Next, we prepared the serially diluted standards from HT-29 EVs whose initial counts (750 × 10^7^) were determined using commercial EXOCET exosome quantification kit (Additional file 1: Figure S1) and created a calibration curve by measuring the FP values after incubating the prepared EV standards with C12-FAM. As shown in Fig. 2a, the FP signal change, ΔFP=FP-FP_0_, where FP_0_ and FP are the respective FP in the absence and presence of EVs increased with increase in the concentration of EVs and exhibited an excellent linear relationship (R^2^ = 0.99) [26, 27]; the limit of detection (3σ/slope) was calculated ca. 28 × 10^7^ EVs (17.5 × 10^5^ EVs/μL), which is comparable or superior to those of other EV quantification methods [28–32]. In order to confirm our assumption that the FP values of C12-FAM are enhanced by the interaction of the lipophilic tail with the EVs, the control dye, FAM that does not have alkyl groups was employed. As envisioned, the control dye generated almost constant ΔFP regardless of the number of EVs (*P* = 0.7775, one-way analysis of variance (ANOVA)). These results were supported by fluorescence microscopy analysis, which clearly confirmed that EVs are stained by C12-FAM, not control FAM dye (Fig. 2b, c) [30, 33, 34]. In addition, the incubation time between C12-FAM and EVs was optimized. The results in Additional file 1: Figure S2 demonstrate that ΔFP increases with increasing incubation time up to 20 min, over which it reaches a plateau. Overall, these observations prove that lipophilic fluorescein probe, C12-FAM binds to EVs with a concomitant increase in the FP values, which can be used for the simple quantification of EVs.

### Accuracy of the developed system

Next, we evaluated the accuracy of the new system by quantifying HT-29 EVs. As shown in Table 1, the counts of HT-29 EVs were determined with excellent precision and reproducibility as evidenced by a coefficient of variation (CV) less than 10% and a recovery ratio between 95 and 102%. In addition, EVs secreted from normal cells, TCMK-1 were also quantified to check the universal applicability of the developed method. Similar to the HT-29 EVs, TCMK-1 EVs were first characterized by SEM and DLS analyses. They were similar to the HT-29 EVs in shape and size (ca. 200 nm) (Additional file 1: Figure S3). Importantly, the concentrations of TCMK-1 EVs were determined with great precision and reproducibility as evidenced by a CV less than 9% and a recovery ratio between 95 and 105% (Additional file 1: Table S1), clearly confirming that the new FP-based system has the potential to reliably determine the EV counts. In addition, it was confirmed that the chemical precipitants have no deleterious effect on the measurement of EVs (Additional file 1: Figure S4). These results are supported by the fact that the proprietary polymer in Exo-Quick-TC that precipitates EVs dissolves when the supernatant is removed and EVs are resuspended in water or PBS [19].

**Table 1:** The accuracy of FP-based quantification method with HT-29 EVs

Sample	Added EV counts (× 10^7^)	Measured EV counts (× 10^7^)	SD^a^	CV (%)^b^	Recovery (%)^c^
A	248	247	18.7	7.6	99.7
B	360	343	12.4	3.6	95.3
C	550	558	54.2	9.7	101.5
D	760	765	3.6	0.5	100.7

^a^Standard deviation of three measurements

^b^Coefficient of variation (%) = SD/mean × 100

^c^Recovery (%) = Measured value/added value × 100

### Detection feasibility of cancer diagnosis

**Fig. 3:**
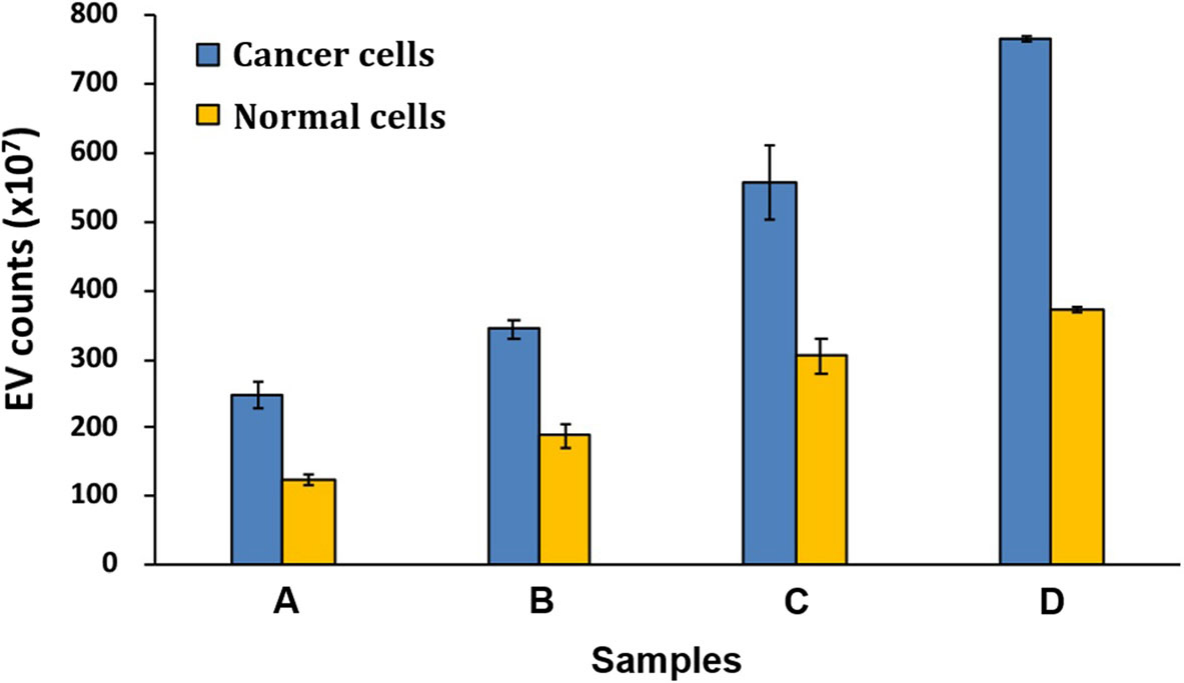
Feasibility of cancer diagnosis. The EVs were isolated from cancer (HT-29) and normal (TCMK-1) cells and quantified by the new FP-based method. The samples (A-D) are equivalent to 3.3 × 10^6^, 5.0 × 10^6^, 7.5 × 10^6^ and 1.0 × 10^7^ cells, respectively

Finally, we investigated the detection feasibility of our method to diagnose cancers by measuring the EV counts instead of specific tumor biomarkers [35, 36]. To demonstrate this possibility, we prepared EVs from two cell lines (cancer cells: HT-29 and normal cells: TCMK-1) at the same cell number. As the results in Fig. 3 show, the cancer cells secreted more EVs than normal cells in a 2-fold increased rate (*P* < 0.0268, two-tailed t-test), which were accurately determined by our FP-based method. In addition, we isolated EVs from serum and quantified EVs with both our FP method and EXOCET. The new results in Additional file 1: Figure S5 show that the developed system successfully quantifies EVs derived from serum, which matches well with that obtained by EXOCET (*P* = 0.8749, two-tailed t-test).

## Conclusion

In summary, we developed a new method for rapid quantification method for EVs based on FP techniques. The new strategy is simple and cost-effective (see Additional file 1: Table S2 in the supporting information for comparison with a commercial kit). It can be performed in less than 20 min and only requires the incubation of EVs with C12-FAM without any washing steps. As a proof-of-concept, EVs from two cell lines, HT-29 and TCMK-1 were successfully quantified with high precision and reproducibility, which is comparable to that of the commercial kit. Importantly, it was demonstrated that the total EV counts could be utilized for the discrimination of cancer from normal cells with the new FP-based method. As compared to NTA that can count individual EVs with the different sizes, the developed method has some drawbacks in that it can give the approximate estimation of EV counts averaged from the population of heterogenous vesicles. However, the size of EVs isolated from the commercial kit is almost similar (ca. 200 nm) as confirmed by the characterization of EVs in Fig. 1 and Additional file 1: Figure S3. In addition, the proposed system aims at the general laboratory that is not equipped with expensive NTA instrument and thus it will be very effective to the users who want to estimate EV counts at the low cost. We expect that the developed system could be universally applied for the quantification of EVs in all biological fluids, including blood, urine, saliva, and breast milk, and would pave the way for the development of a simple and rapid tool for early diagnosis of cancers.

## Materials and methods

### Materials

5-dodecanoylamino fluorescein (C12-FAM) and fluorescein were purchased from Thermo Fisher Scientific (USA) and Sigma–Aldrich (USA), respectively. Exosome-depleted fetal bovine serum (FBS), ExoQuick-TC exosome precipitation solution and EXOCET exosome Quantitation kit were purchased from System Biosciences (USA). Dulbecco’s modified Eagle’s medium (DMEM), and penicillin/streptomycin were purchased from Gibco BRL (USA). FBS and Macrosep Advance Centrifugal Devices (30 kDa) were purchased from Youngin frontier (Korea) and Pall Corporation (USA), respectively. Aqueous solutions were prepared using ultrapure DNase/RNase-free distilled water (D.W.) purchased from Bioneer. All other chemicals were of analytical grade and used without further purification.

### Cell culture

HT-29 (KCLB, 30038) and TCMK-1 (KCLB, 10139) cells were grown in DMEM medium supplemented with 10% FBS, 100 U/mL penicillin and 100 μg/mL streptomycin at 37 °C in a humidified atmosphere of 5% CO_2._

### Isolation of EVs

The EVs were isolated from cells and serum using ExoQuick-TC exosome precipitation solution according to the manufacturer’s protocol. Briefly, cells were grown for 48 h in exosome-depleted medium (with 5% exosome-depleted FBS). Conditioned medium was collected and centrifuged at 1500 g for 15 min to remove cells and debris. The media supernatant was then concentrated through a 30 kDa filter and transferred to a new tube and mixed with ExoQuick-TC exosome precipitation solution. After incubation at 4 °C overnight, the mixture was centrifuged at 1500 g for 30 min. The pellet that was formed at the bottom of the tube was resuspended in phosphate-buffer saline (PBS).

### Characterization of EVs

SEM images were obtained by using Field Emission Scanning Electron Microscope (HITACHI SU8010, Hitachi Corporation, Japan). For the preparation of samples, the EVs were first fixed with 100% methanol (Sigma–Aldrich, USA) at − 20 °C for 20 min. Next, the fixed EVs were washed twice with PBS and then dehydrated with ascending concentrations of ethanol (50, 70, 80, and 95%) [37]. After the complete removal of ethanol, the samples were left to dry at room temperature and then analyzed after platinum coating. For measuring size distribution, the EVs dissolved in PBS were analyzed using dynamic light scattering (DLS) (DynaPro Plate Reader, Wyatt Technology, USA). The size of EVs were analysed by number of percent (Z average) at a fixed angle using software provided by the instrument. For fluorescence microscopy imaging, EVs were first incubated with the C12-FAM at 1.6 μM for 20 min and then dropped on the glass slide. The resulting images were obtained by fluorescence microscopy (Olympus BX51(Japan) equipped with ACD see 5.0).

### EXOCET-based quantification of EVs

The isolated EVs were quantified using EXOCET exosome quantification kit according to the manufacturer’s protocol. Briefly, after mixing the EXOCET reaction buffer with the lysed EVs, the solution was incubated for 20 min at room temperature. The absorbance was measured at a wavelength of 405 nm (SpectraMax iD5 multi-mode microplate reader, Molecular Devices, USA).

### FP-based quantification of EVs

The isolated EVs were mixed with 1.6 μM C12-FAM in a reaction buffer composed of 1 mM HEPES (pH 8) and 1.6 mM NaCl in a total reaction volume of 160 μL. After the incubation of the reaction mixture for 20 min at room temperature, the fluorescence polarization values were measured at the excitation and emission wavelengths of 485 and 528 nm, respectively (SpectraMax iD5 multi-mode microplate reader, Molecular Devices, USA). The concentration of C12-FAM was determined 1.6 μM because it is suggested that lipophilic fluorescent dye should be used at the concentration less than 2 μM for the most reproducible results.

### Accuracy confirmation of the developed system

The isolated EVs were split into two, which were quantified by EXOCET and FP-based EV quantification methods, respectively. The added and measured EV counts in Table 1 and Additional file 1: Table S1 were measured by EXOCET and FP-based methods, respectively, according to the procedures explained above. In both cases, the calibration curves were first created with a set of standards containing known EV counts, and the EV counts of unknown samples were determined from the calibration curve.

## Additional file

Additional file 1: **Figure S1.** The standard curve obtained from EXOCET exosome quantification kit. **Figure S2.** The optimization of incubation time between C12-FAM and EVs. **Figure S3.** Characterization of TCMK-1 EVs. (A and B) SEM image of EVs. (C) Size distribution of EVs. **Figure S4.** The effect of chemical precipitant for the accurate quantification of EVs. 1 and 2 indicate the samples for EVs + C12-FAM and EVs + C12-FAM + Exoquick precipitation solution, respectively. The number of EVs is 6.5 × 10^9^/mL. **Figure S5.** The quantification of EVs isolated from serum. EVs isolated from serum were split into two, which were measured by our FP method (1) and EXOCET (2), respectively. **Table S1.** The accuracy of FP-based EV quantification with TCMK-1 EVs. **Table S2.** Comparison of our method with the commercial one. (DOC 145 kb)

## Abbreviations

AChE: Acetylcholinesterase
C12-FAM: 5-dodecanoylamino fluorescein
DLS: Dynamic light scattering
EVs: Extracellular vesicles
FP: Fluorescence polarization
iMER: immuno-magnetic exosome RNA
NTA: Nanoparticle tracking analysis
SEM: Scanning electron microscopy
