# Supplementary material for: A simple approach for rapid and cost-effective quantification of extracellular vesicles using a fluorescence polarization technique
Source: J Biol Eng. 2019 Apr 16;13:31. doi: 10.1186/s13036-019-0160-9 (PMC6469078; doi:10.1186/s13036-019-0160-9)
Supplement: Supplementary file 1 — Figure S1. The standard curve obtained from EXOCET exosome quantification kit. Figure S2. The optimization of incubation time between C12-FAM and EVs. Figure S3. Characterization of TCMK-1 EVs. (A and B) SEM image of EVs. (C) Size distribution of EVs. Figure S4. The effect of chemical precipitant for the accurate quantification of EVs. 1 and 2 indicate the samples for EVs + C12-FAM and EVs + C12-FAM + Exoquick precipitation solution, respectively. The number of EVs is 6.5 × 10^9^/mL. Figure S5. The quantification of EVs isolated from serum. EVs isolated from serum were split into two, which were measured by our FP method (1) and EXOCET (2), respectively. Table S1. The accuracy of FP-based EV quantification with TCMK-1 EVs. Table S2. Comparison of our method with the commercial one. (DOC 145 kb) [file 13036_2019_160_MOESM1_ESM.doc]

Supporting information for

**A simple approach for rapid and cost-effective quantification of extracellular vesicles using a fluorescence polarization technique**

Kalishwaralal Kalimuthu,^#^ Woo Young Kwon,^#^ and Ki Soo Park*

Department of Biological Engineering, College of Engineering, Konkuk University, Seoul 05029, Republic of Korea

^#^ These authors equally contributed to this work

*Address for correspondence:

Prof. Ki Soo Park

Assistant Professor,

Department of Biological Engineering,

College of Engineering,

Konkuk University,

Seoul, 05029 Republic of Korea.

E.mail: kskonkuk@gmail.com or akdong486@konkuk.ac.kr

**
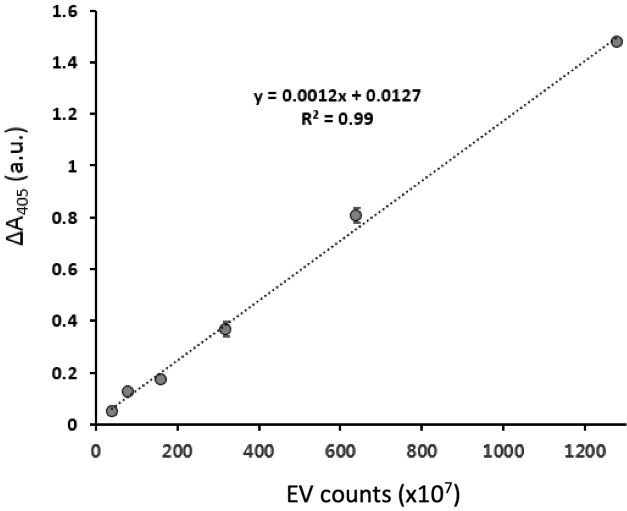
**

**Figure S1.** The standard curve obtained from EXOCET exosome quantification kit.

**
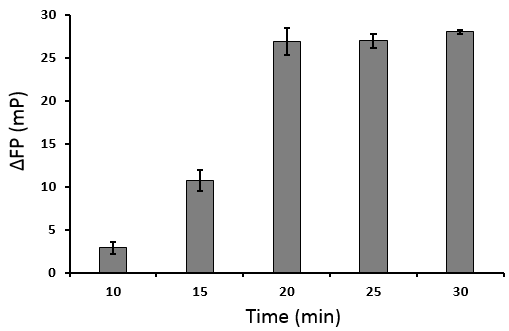
**

**Figure S2.** The optimization of incubation time between C12-FAM and EVs.

**
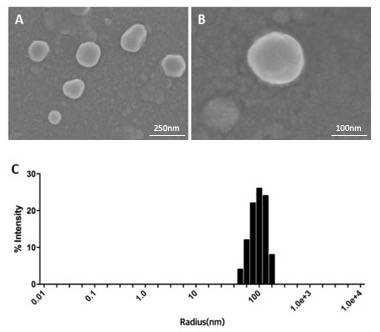
**

**Figure S3.** Characterization of TCMK-1 EVs. (A and B) SEM image of EVs. (C) Size distribution of EVs.

**
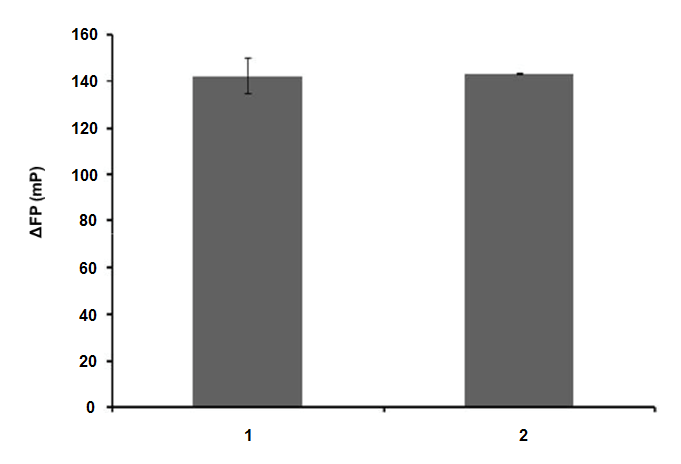
**

**Figure S4.** The effect of chemical precipitant for the accurate quantification of EVs. 1 and 2 indicate the samples for EVs + C12-FAM and EVs + C12-FAM + Exoquick precipitation solution, respectively. The number of EVs is 6.5 x 10^9^/mL.

**
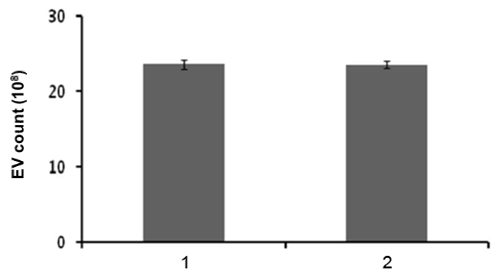
**

**Figure S5**. The quantification of EVs isolated from serum. EVs isolated from serum were split into two, which were measured by our FP method (1) and EXOCET (2), respectively.

**Table S1.** The accuracy of FP-based EV quantification with TCMK-1 EVs.

| **Sample** | **Added EV counts (x10^7^)** | **Measured EV counts (x10^7^)** | **SD^[a]^** | **CV (%)^[b]^** | **Recovery(%)^[c]^** |
| --- | --- | --- | --- | --- | --- |
| A | 130 | 124 | 8.4 | 6.8 | 95.1 |
| B | 179 | 189 | 16.7 | 8.9 | 105.4 |
| C | 310 | 305 | 25.4 | 8.3 | 98.4 |
| D | 365 | 370 | 3.3 | 0.9 | 101.5 |

[a] Standard deviation of three measurements.

[b] Coefﬁcient of variation = SD/mean × 100.

[c] Measured value/added value × 100

**Table S2.** Comparison of our method with the commercial one.

|  | **Our method** | **EXOCET exosome quantification kit** |
| --- | --- | --- |
| **Principle** | Fluorescence polarization detection of lipophilic fluorescence probe | Colorimetric detection of acetylcholinesterase activity |
| **Sensitivity** | 17.5 x 10^5^ EVs/μL | 28.3 x 10^5^ EVs/μL |
| **Assay time** | 20 min | 30 min |
| **Cost** | 0.01$ for a single assay | 6$ for a single assay |
